# Jets and Mirror Mode Waves in Earth's Magnetosheath

**DOI:** 10.1029/2022JA031221

**Published:** 2023-07-22

**Authors:** X. Blanco‐Cano, D. Rojas‐Castillo, P. Kajdič, L. Preisser

**Affiliations:** ^1^ Instituto de Geofísica Universidad Nacional Autónoma de México Circuito de la Investigación Científica s/n Ciudad Universitaria Mexico City Mexico; ^2^ Space Research Institute Austrian Academy of Sciences Graz Austria

**Keywords:** magnetosheath jets, mirror mode, micro‐physics, ion distribution

## Abstract

Magnetosheath jets are localized plasma structures with high dynamic pressure which are frequently observed downstream of the Earth's bow shock. In this work we analyze Magnetospheric MultiScale magnetic field and plasma data and show that jets can be found in the quasi‐perpendicular magnetosheath in regions permeated by Mirror mode waves (MMWs). We show that structures identified as jets by their enhanced dynamic pressure can have very different internal structure, with variable signatures in magnetic field magnitude and components, velocity, and density and can be associated to ion distribution functions of various types. This suggests that jets observed in the quasi‐perpendicular magnetosheath are generated by different mechanisms. We find that jets can be related to traveling foreshocks, flux transfer events, and some have MMWs inside them. Our results suggest that some jets have a local source and their formation does not depend on upstream structures. We find that different types of ion distributions can exist inside the jets, while in some cases anisotropic distributions are present, in others counterstreaming distributions exist. We also show that for jets with MMWs inside them, ion distributions can be modulated. This highlights the importance of using ion distributions to identify and classify different types of jets.

## Key Points

- We study high speed magnetosheath jets coexisting with mirror mode waves
- We use Magnetospheric MultiScale data to show that high speed magnetosheath jets have a much more complex structure than previously thought. Velocity distribution functions can be a tool to describe the micro‐physics inside jets
- High speed jets in the quasi‐perpendicular magnetosheath could have different generation mechanisms that those in the quasi‐parallel sheath

## 1 Introduction

The study of jets has become one of the most important subjects of magnetosheath research in recent years. These jets are structures with dynamic pressure (P_*dyn*_) values above those in the upstream solar wind (SW) (Plaschke et al., 2018), or above those in their surrounding magnetosheath (Archer & Horbury, 2013). Such a located increment in *P*_*dyn*_ can be due to enhancement of the magnetosheath plasma speed, density, or a combination of both. Jets have been mostly found in the quasi‐parallel magnetosheath (Archer & Horbury, 2013; Němeček et al., 1998; Plaschke et al., 2013; Vuorinen et al., 2019), the region downstream of the quasi‐parallel bow shock where *θ*_*Bn*_ < 45° (*θ*_*Bn*_ is the angle between the upstream magnetic field vector and the shock normal). Therefore suggesting that their origin is linked to foreshock and bow shock phenomena (Hietala et al., 2012; Plaschke et al., 2018). However, jet origin is still an unsolved question opening the possibility for jets to be generated by different mechanisms (Plaschke et al., 2018). The most accepted ones are that jets are: (a) generated at the quasi‐parallel shock due to its inherent rippled surface (Hietala et al., 2009; Plaschke et al., 2013; Preisser, Blanco‐Cano, Kajdič, et al., 2020, Preisser, Blanco‐Cano, Trotta, et al., 2020), (b) due to the interaction of interplanetary magnetic field (IMF) discontinuities with the bow shock (Archer et al., 2012), (c) due to hot flow anomalies (HFAs, Savin et al., 2012) or (d) due to transmission of short large amplitude magnetic structures (SLAMS) or SW plasmoids into the magnetosheath (Karlsson et al., 2012, 2015, 2016), (e) due to a combined effect of upstream plasma wave evolution and an ongoing reformation cycle of the bow shock (Raptis, Karlsson, Vaivads, Pollock, et al., 2022).

Recent statistical studies have favored the bow shock ripple mechanism and the SLAMS mechanism (Raptis et al., 2020). Hietala and Plaschke (2013) showed that when the angle between the IMF and the Sun–Earth line is small, 97% of the jets observed near the bow shock can be produced by local shock ripples. New simulation results (Suni et al., 2021) showed that up to 75% of the jets observed downstream of the quasi‐parallel bow shock are caused by foreshock compressive structures favoring the SLAMS jet association.

A number of past studies have determined some of the jets' properties reporting jet scale sizes of the order of ∼1 R_*E*_ (Archer et al., 2012; Plaschke et al., 2016; Savin et al., 2012). However, in a more recent analysis of jets in the subsolar region Plaschke et al. (2020) found that typical jet scales can be much smaller, around 0.1*R*_*E*_. Such jets have average duration of ∼30 s. On the other hand the existence of large scale jets with durations from 30 s to 3 min peaking at 80 s has been shown by Dmitriev et al. (2021). These authors show that in contrast to the smaller scale jets which occur predominantly in the subsolar region, the large jets have been observed in a wide longitudinal range of ∼70°, which can include the quasi‐perpendicular magnetosheath.

We still know little about jet micro‐structure, and how they interact with the surrounding plasma and the waves in it. Plaschke et al. (2017) showed that the internal structure of jets can be very rich, with large amplitude density, temperature, and magnetic field variations over small scales/short periods of time. Karlsson et al. (2018) showed that jets can be associated with whistler, lower hybrid, and broadband electrostatic waves, as well as with compressive electromagnetic waves with periods of 10 s. Wave amplitudes are higher within the jets than in the surrounding quasi‐parallel magnetosheath. These authors also found that jets occurring at the transition region between the quasi‐parallel (*Q*_‖_) and quasi‐perpendicular (*Q*_⊥_) magnetosheath (the regions downstream of the quasi‐parallel and quasi‐perpendicular bow shock, respectively) can exhibit ion populations with VDFs similar to those observed in both, *Q*_‖_ and *Q*_⊥_ magnetosheaths.

In a previous work, Blanco‐Cano et al. (2020) found that jets can be observed in regions permeated by mirror mode waves (MMWs) while the waves inside jets have larger transverse components, which is in contrast to the compressive character of the MMWs. It was also found that jets can be associated with different types of VDFs. While some jets are associated to isotropic VDFs, others have two field aligned ion beams inside them.

Vuorinen et al. (2019) found that jets are observed nine times more often downstream of the quasi‐parallel bow shock than of the quasi‐perpendicular shock. Most studies have focused on jets downstream of the quasi‐parallel shock, therefore we have a rather limited knowledge about their characteristics in the quasi‐perpendicular magnetosheath. Raptis et al. (2020) found that jets in the quasi‐perpendicular magnetosheath exhibit a much smaller dynamic pressure than their counterparts downstream of the quasi‐parallel shock. Kajdič et al. (2021) showed that jets observed in the quasi‐perpendicular magnetosheath can be related to (a) magnetic flux tubes connected to the quasi‐parallel bow‐shock (the downstream equivalents of traveling foreshocks), (b) non‐reconnecting current sheets, (c) reconnection exhausts, and (d) isolated MMWs. Such jets are thus not caused by shock ripples. In this work we show that jets in the quasi‐perpendicular magnetosheath can also be related to flux transfer events (FTEs), and can contain or be surrounded by trains of MMWs.

Understanding jets internal structure and their evolution through the magnetosheath is important because they can impact the magnetopause causing local indentations in the magnetosphere, and can launch surface waves (Archer et al., 2019) or compressional waves (Plaschke et al., 2009). It has been shown that the arrival of jets to the magnetopause can also trigger local magnetic reconnection (Hietala et al., 2018; Ng et al., 2021), and can result in entrance of plasma into the magnetosphere (Dmitriev & Suvorova, 2015; Gunell et al., 2014; Karlsson et al., 2012). Ionospheric flow enhancements have been also associated to jets (Hietala et al., 2012; Ng et al., 2021), and a study by Han et al. (2017) has suggested that dayside auroras known as “throat” auroras may be caused by jet interaction with the magnetopause. In a recent study, Wang et al. (2022) reported that jets can trigger ultra low frequency ground waves. It is expected that jets occur at other planetary and astrophysical bow shocks, see for example, Karlsson et al. (2016) who studied jets in Mercury's environment. Therefore, understanding their properties near Earth gives insight about their properties in other environments.

Although jets have been studied for close to 20 years it is clear that many of their detailed properties are not well established, and are based on relatively few case studies. Jets come in different “flavors” (structures/morphology) not only because of the diverse selection criteria, but also because of their very different origins. They can be observed in regions where other magnetosheath phenomena, such as MMWs exist and we know little about their interaction. MMWs are very compressive with anti‐correlated fluctuations in magnetic field magnitude and density. Thus it is possible that plasma parcels with jets and MMWs can have a strong impact on the magnetopause.

From all the aforementioned, the aim of this work is to investigate jets properties in regions with MMWs and the way they interact with such waves. We find that jets observed within regions permeated by MMWs can have very different morphologies, for instance a clear magnetic field rotation can occur inside some of them leading to interesting configurations in ion pitch angle and diverse velocity ion distributions. Our results suggest different origin for the analyzed jets. We also investigate the characteristics of waves observed inside the jets and in the surrounding regions. The following section presents case studies of jets, waves and ion distributions, followed by a discussion and conclusion section.

## 2 Case Studies

We use data from the Magnetospheric Multiscale Mission (Burch et al., 2016). Plasma data are from the Fast Plasma Instrument (FPI) (Pollock et al., 2016) with a time resolution of 4.5 s and the magnetic field data with 16 samples/s from the Fluxgate Magnetometer (Russell et al., 2016).

A number of criteria have been used in the literature to identify magnetosheath jets (see Table 1 in Plaschke et al. (2018)). In this work we use the criteria of Archer and Horbury (2013), which states that the dynamic pressure inside the jets has to reach values of at least twice the background magnetosheath dynamic pressure.

**Figure 1:**
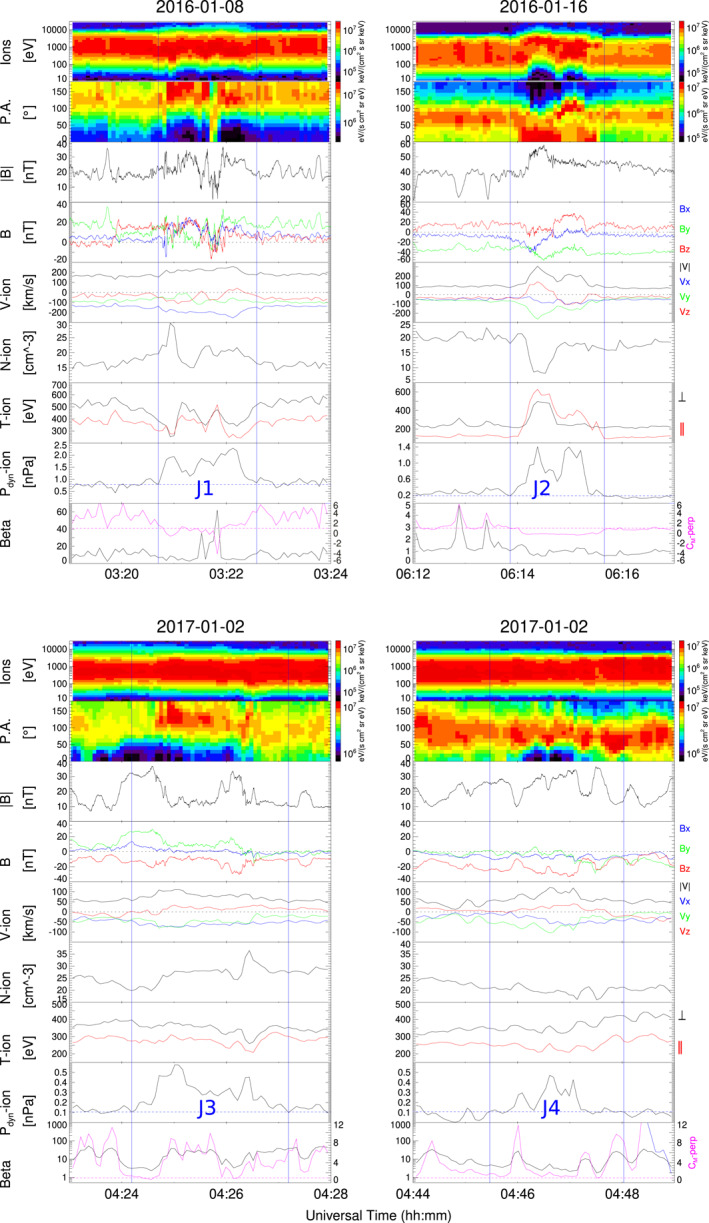
MMS‐1 data showing the properties of the four jets in this study. From top to bottom panels show ion energy spectra, pitch angle spectra, magnetic field magnitude and components, velocity magnitude and components, ion density, parallel and perpendicular ion temperature, dynamic pressure, plasma beta (black) and mirror instability threshold (magenta). Jet intervals are delimited by blue vertical lines. The horizontal line in the *P*_*dyn*_ panel indicates the value of $2*<{P_{\mathrm{dyn}}>}_{BG}$.

Figure 1 shows the four jets studied in this work. From top to bottom the panels show ion energy spectra, ion pitch angle (for ions with energies ranging from 10 eV to 26 keV), magnetic field magnitude (B) and components (in geocentric solar ecliptic coordinates), velocity magnitude and components, ion density (N), parallel and perpendicular ion temperature, dynamic pressure (*P*_*dyn*_), plasma beta (the ratio between the thermal pressure and the magnetic pressure) in black and mirror instability threshold (magenta) *C*_*M*_ − *perp* = *β*_⊥_(*T*_⊥_/*T*_‖_ − 1) (A. Hasegawa, 1969; Génot et al., 2009). Identified jet intervals are labeled J1, J2, J3, and J4 and delimited by blue vertical lines. All four jets propagate anti‐sunwards with negative *V*_*x*_, and are observed in the quasi‐perpendicular magnetosheath with *T*_⊥_/*T*_‖_ > 1 (see Figure 1 of Kajdič et al. (2021)). By looking only at the profiles of *P*_*dyn*_ all jets look very similar with increments that last 85–163 s. However, as we will describe below, other properties differ from jet to jet, suggesting different origins. Jets observed on 8 January 2016 and 16 January 2016 (J1 and J2 respectively) show significant rotation in the magnetic field. In the case of J2 this rotation is bipolar in *B*_*x*_. The ions inside them reach higher energies in comparison with their surroundings, so at first glance they could be classified as encapsulated following the classification of Raptis et al. (2020). In contrast, jets observed on 2 January 2017 (J3 and J4) show no field rotation, occurred in regions with predominantly *B*_*z*_ south, and would be classified as quasi‐perpendicular according to Raptis et al. (2020). In all the four cases the jets were preceded and followed by regions with MMWs, with different characteristic as we describe further down.

**Figure 2:**
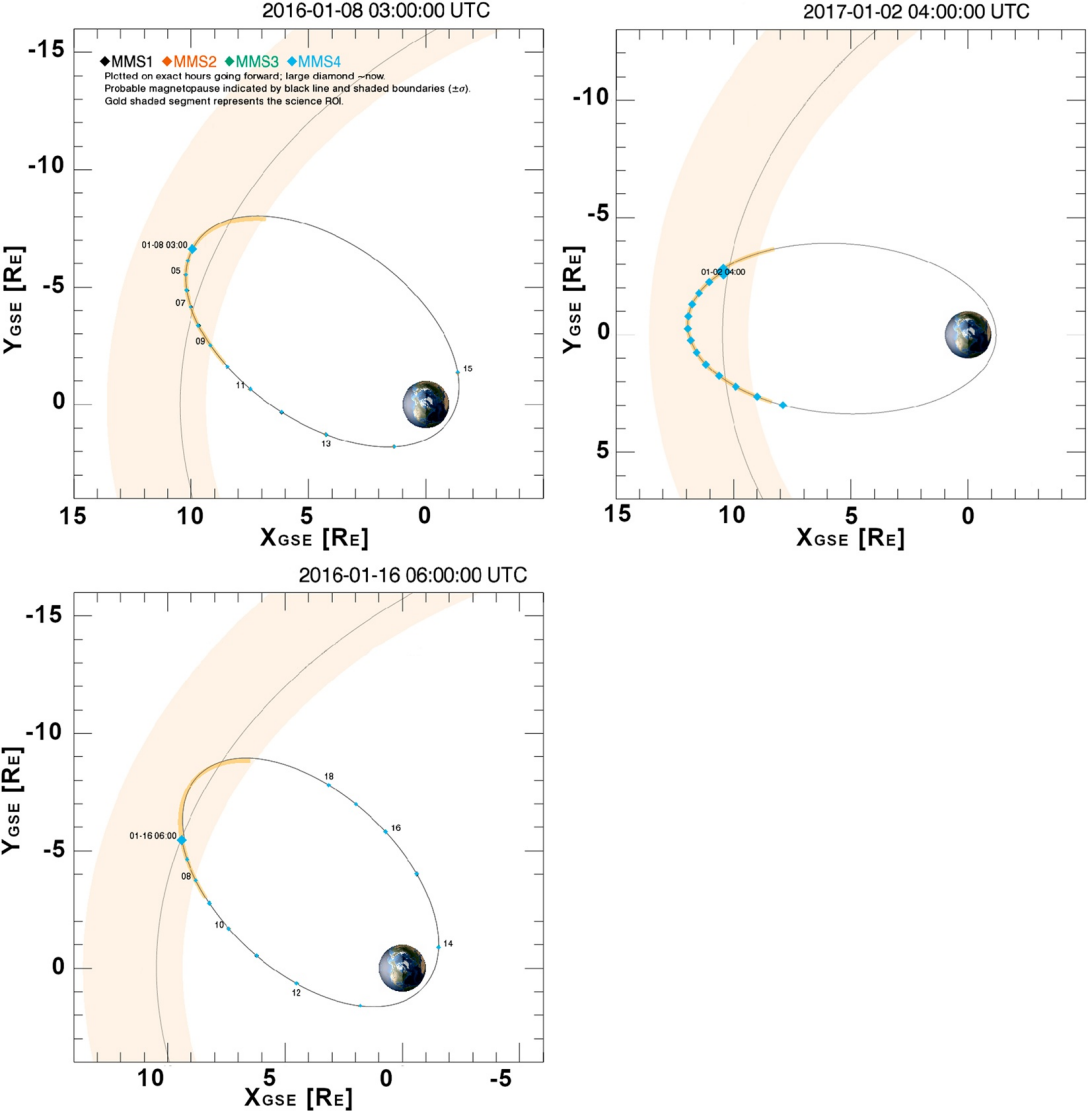
Magnetospheric MultiScale orbits on 8 January 2016, 16 January 2016, and 2 January 2017 at the times of jet observations. The black line inside the orange strip represents the modeled and probable location of the magnetopause respectively. Panels are shown in the XY plane in *R*_*E*_ units.

Figure 2 shows the orbits of the MMS tetrahedron at the times of the observations and the location of the nominal magnetopause provided by https://lasp.colorado.edu/mms/sdc/public/ using the model of Shue et al. (1998). The solid black line indicates the mean magnetopause and shaded regions indicate the range of probable magnetopause locations. In all the cases the jets were closer to the magnetopause than to the bow shock. In fact, the jets observed on 16 January 2016 and 2 January 2017 were just outside of the mean magnetopause location given by the model.

### 2.1 Jet Within a Mirror Mode Storm

**Figure 3:**
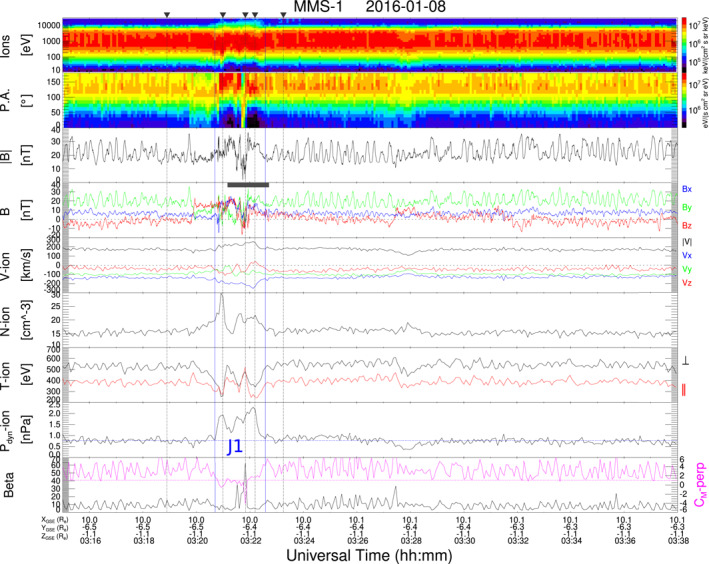
MMS‐1 data on 8 January 2016 showing J1 and an extended region with mirror mode waves. Panels are in the same format as Figure 1. Vertical dashed lines indicate the times of ion distributions on Figure 4. The solid horizontal line indicates the intervals corresponding to the Fourier analysis displayed on Figure 11.

Figure 3 shows MMS‐1 data during an interval on 8 January 2016 that contains J1. The jet is observed just after the magnetic field component *B*_*z*_ changed from southward to northward. J1 shows a clear increment in dynamic pressure (*P*_*dyn*_) due to enhancements of velocity (*V*) and density (*N*) during 110 s. The ion energy spectra suggests that the plasma inside the jet has characteristics of the quasi‐parallel magnetosheath that is, with a broader energy spectra reaching energies of ∼20 keV, while the surrounding plasma indicates a quasi‐perpendicular magnetosheath with an ion energy spectra of lower energies and a sustained temperature anisotropy with *T*_⊥_ > *T*_‖_. From the ion energy spectra this jet could be classified as encapsulated following the classification of Raptis et al. (2020). However, the drop in temperature anisotropy within J1 only occurs in two short intervals and not throughout the whole jet as expected for plasma of the quasi‐parallel magnetosheath, so the plasma in the jet shows characteristics of both, quasi‐parallel and quasi‐perpendicular magnetosheath. The magnetic field inside J1 has a complex structure with (a) a rotation in all components, (b) compressive fluctuations and (c) a decrement of field magnitude of around 80% at the jet core. The occurrence of large compressive fluctuations at the times where temperature anisotropy drops suggests that these electromagnetic fluctuations contribute to isotropize the plasma. It is interesting to note that not all these field fluctuations are in phase with the density changes, and the field rotation causes changes in ion pitch angle inside J1 (see also Figure 1). In addition, the value of *C*_*M*_ − *perp* drops within J1, so the plasma is not unstable to mirror mode. In contrast, mirror mode waves (MMWs, characterized by B and N oscillations in anti‐phase) are observed before and after the jet, where *C*_*M*_ − *perp* values >1. The MMWs permeate a large and continuous (∼2 hr) region of the magnetosheath, and can be classified as a mirror mode storm (Russell et al., 2009). The magnetic field rotations bounding the jet suggest that the origin of J1 is related to a traveling foreshock which forms upstream due to a SW discontinuity that changes the IMF configuration (Kajdič et al., 2017).

**Figure 4:**
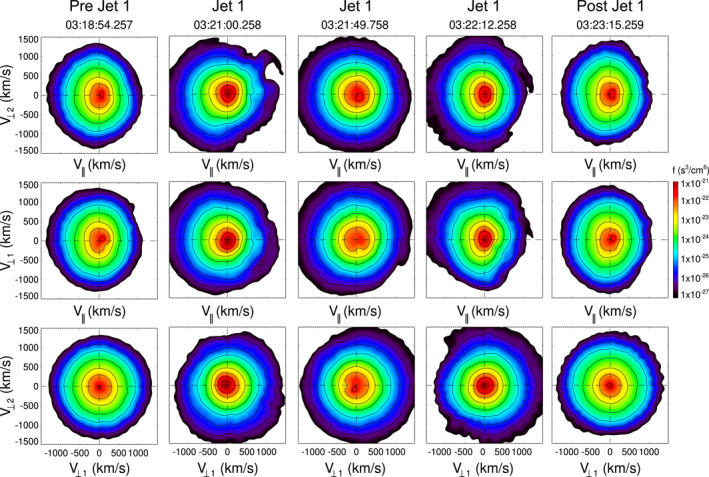
Ion distributions associated with J1, and surrounding regions. Distributions are given for three planes, where *V*_⊥1_, *V*_⊥2_, and *V*_‖_ indicate directions perpendicular and parallel to the *B*‐field.

We use fast survey mode data with a resolution of 4.5 s from the FPI instrument to investigate the characteristics of ion distributions through the intervals of study. Figure 4 shows ion VDFs inside J1 and near it (see the dashed vertical lines in Figure 3). The labels *V*_⊥1_, *V*_⊥2_, and *V*_‖_ indicate directions perpendicular and parallel to the *B*‐field.

The VDFs in the regions pre and post J1 (03:18:54.257 and 03:23:15.259 UT) have a bi‐Maxwellian shape with a *T*_⊥_ > *T*_‖_, typical of the quasi‐perpendicular (*Q*_⊥_) magnetosheath. In contrast, some of the VDFs inside J1 (03:21:00.258 UT) can be more isotropic, others (03:21:49.758 UT) show a spread along *V*_‖_ with positive and negative values, which coincides with the changes in pitch angle shown in Figure 3. At the rear part of J1 (03:22:12.258 UT) VDFs are again anisotropic, with *T*_⊥_ > *T*_‖_.

### 2.2 Jet With a Double Structure

**Figure 5:**
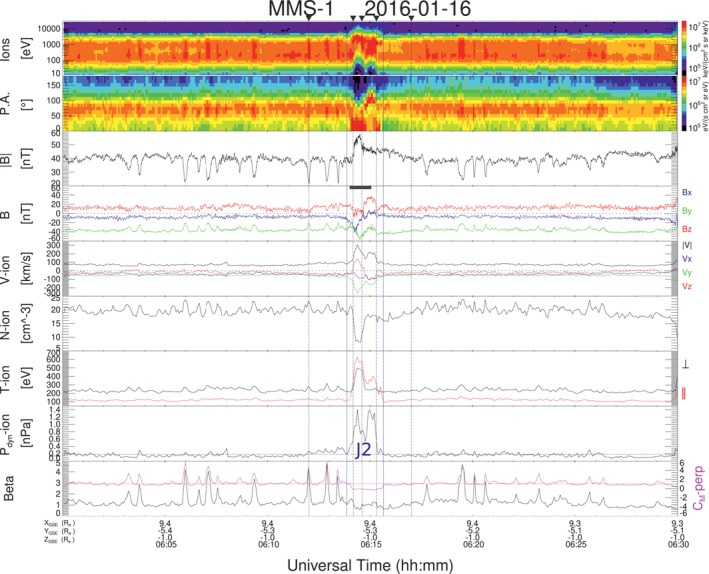
MMS‐1 data on 16 January 2016 showing and interval with a jet immersed in a region with mirror mode waves. The format is as in Figure 1.

Figure 5 shows an interval on 16 January 2016 where MMS‐1 observed J2 surrounded by MMWs. The jet region can be clearly identified from the *P*_*dyn*_ enhancement and it lasts for 85 s. This event has a double peak in the profiles of *P*_*dyn*_, *V* and *T*_‖_, which indicates that the jet is formed by two substructures: J2/1, the leading structure (06:13:51‐06:14:55) is fast with rarefied plasma, *V*_*z*_ > 0, and enhanced field magnitude; J2/2, the second structure (0614:55‐0615:48) is fast and dense with *V*_*z*_ < 0.

Inside J2/1, the magnetic field shows a clear rotation in *B*_*x*_. The variations of density and magnetic field magnitude inside this structure suggest a mirror mode, however the instability criteria *C*_*M*_ − *perp* is not satisfied within this short interval. The value of *T*_⊥_ is larger than *T*_‖_ during the whole interval except inside the jet, where not only *T*_‖_ > *T*_⊥_ but the total temperature increases which is in contrast to the other jets studied in this work. As we will show later on (see Figure 7), *T*_⊥_/*T*_‖_ < 1 occurs due to the fact that inside this jet two different ion populations coexist. The ion energy spectra and pitch angle values are also different inside the jet than outside, with the spectra shifted to higher energies and a larger spread in pitch angle. Within J2/1 ions have pitch angle distributions below ∼100°, as opposed to the rear part of the jet where two ion populations exist, one with pitch angle values close to 0°, and the other with values ∼120°.

**Figure 6:**
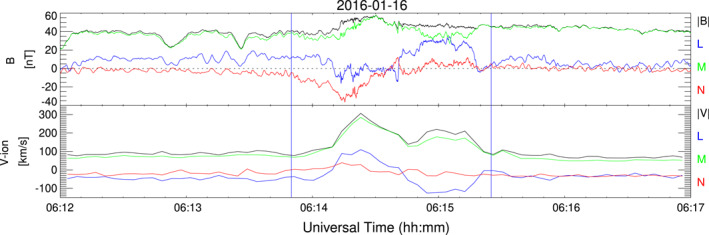
MMS‐1 data on 16 January 2016 showing magnetic field and velocity (magnitude and components) in boundary normal coordinates for an interval including J2 (between blue lines).

Some of the characteristics of J2/1, namely the smooth rotation of the field and the increment in B field magnitude and temperature, suggest that it could be identified as a FTE. In order to verify such a possibility, we plot the magnetic field in boundary normal coordinates, LMN (Russell & Elphic, 1978). To transform MMS magnetic field data to boundary normal coordinates, OMNI data was used as input for the upstream conditions into the Shue et al. (1998) magnetopause model. The resultant LM components lie on the magnetopause tangent plane and the N component is normal to the magnetopause. Here it is worth to point out that J2 is observed at low latitudes below the ecliptic in the dusk sector. From Figure 6 it can be seen that J2/1 has the typical bipolar signature of FTEs in the *B*_*N*_ component: it rotates from negative to positive values as corresponds to a reverse FTE in the southern hemisphere. Such a *B*_*N*_ is a distinctive signature of FTEs but it is not commonly observed inside the jets. The bottom panel of Figure 6 displays velocity magnitude and components in LMN coordinates. It can be seen that there is motion of the two structures that form J2 along the magnetopause in the LM plane. The plasma in which the jet was embedded was propagating in the ‐L direction (southwards), while the leading structure of the jet (J2/1) moved northwards. In the M direction, the structure traveled toward the dawn. In the normal direction the leading part, that is, J2/1 moved away from the magnetopause with a rather low speed.

Thus, J2/1 fulfills simultaneously the characteristics of a magnetosheath jet and of an FTE. Therefore, its origin is linked to magnetic reconnection. However, as shown in Figure 5, *B*_*z*_ was positive during the whole interval and J2 is far from the northern cusp so the *B*_*N*_ rotation as an effect of local reconnection due to negative *B*_*z*_ is not possible. Two possible explanations arise: (a) J2 could be an FTE which moved from its region of origin, (b) J2 might be driven by bursty reconnection which has been shown to occur due to the arrival of jets to the magnetopause (Ng et al., 2021).

Mirror waves with B and N in anti‐phase are also observed before and after J2. In contrast to the MMWs observed near J1, during this interval the MMWs appear as separated holes in the field magnitude instead of more uniform peaks, and *C*_*B*_ − *perp* > 1 only inside the field holes, where the plasma beta is enhanced.

**Figure 7:**
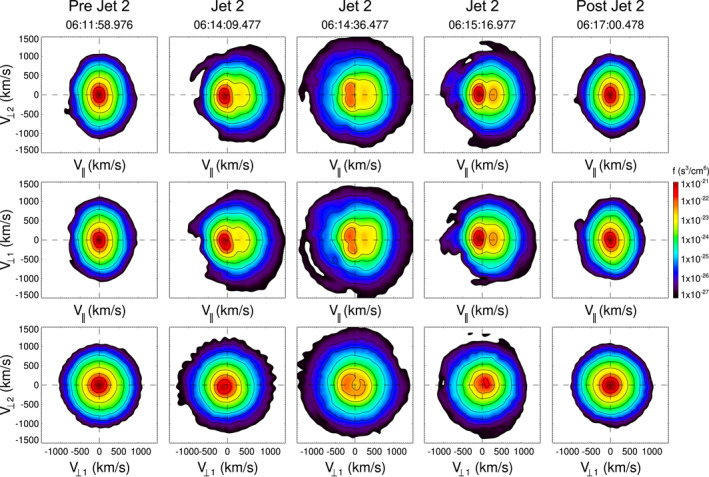
Velocity distribution functions near and inside J2. The format is as in Figure 4.

Figure 7 shows the VDFs before, inside and after J2. The pre and post jet VDFs (06:11:58.976 and 06:17:00.478 UT) show a single ion distribution with *T*_⊥_/*T*_‖_ > 1, while inside J2 two counterstreaming ion populations can be observed. As mentioned above, the temperature inside J2 increases. These plasma properties and particle distributions are similar to two events reported in Blanco‐Cano et al. (2020), but are very different from those reported earlier by other authors (Archer & Horbury, 2013; Archer et al., 2012; Dmitriev & Suvorova, 2012; Karlsson et al., 2018; Plaschke et al., 2013; Shue et al., 2009) whose jets usually exhibit diminished temperature. The VDFs inside J2 are also different from those reported by Karlsson et al. (2018), which exhibit only one ion population. The secondary ion population inside J2/1, with *V*_‖_ > 0 is less dense than inside J2/2 and the surroundings, which supports the fact that J2/1 contains hot less dense magnetospheric plasma as is often the case inside FTEs. At (06:14:36.477 UT) a gyrating ring is clear in the *V*_⊥1_, *V*_⊥2_ plane. The VDFs inside J2/2 also show evidence of two ion populations, one related to incident sheath ions (see e.g., H. Hasegawa et al., 2016; Petrinec et al., 2020), and the other at positive *V*_*x*_. It is interesting to note that the increase in plasma temperature inside J2/2 is related to the presence of the secondary beam, and possibly results from calculating a full moment for the temperature out of the two populations. The perpendicular temperature does not change from the value in the surrounding of the jet. In a recent study Raptis, Karlsson, Vaivads, Lindberg, et al. (2022) showed that jets downstream of the quasi‐parallel bow shock can exhibit two ion populations and showed different ways to obtain separate plasma moments from each particle distribution, which is more accurate to describe double populations than calculating full moments for an all‐in one distribution.

### 2.3 Jet With MMWs Inside

**Figure 8:**
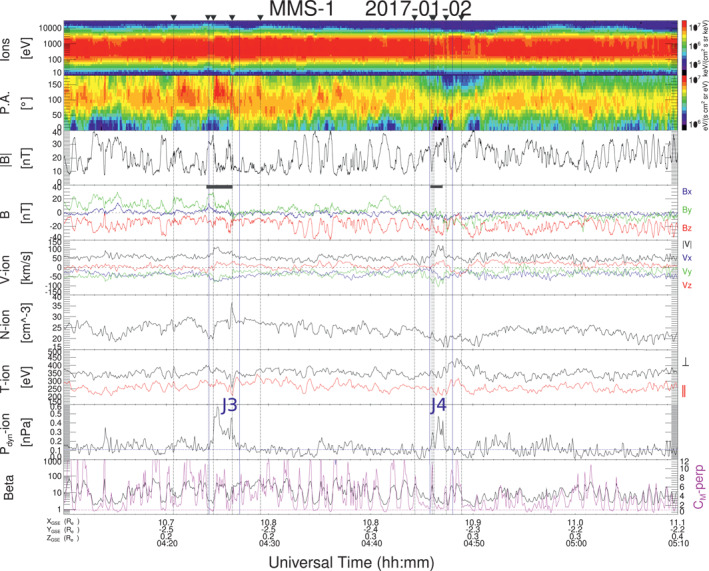
MMS‐1 data on 2 January 2017 showing and interval with two jets J3 and J4 immersed in a region with mirror mode waves. The format is as in Figure 1.

Figure 8 shows an interval with two jets, labeled as J3 and J4, identified by the *P*_*dyn*_ criteria and due mainly to a V increment. In the case of J3 there is also a narrow peak in density at the rear part of the jet. Though these jets (J3 and J4) also occur in the quasi‐perpendicular magnetosheath, as indicated by the energy spectra and the fact that *T*_⊥_ > *T*_‖_ through the shown interval, both jets have very different characteristics to the previous examples. The magnetic field magnitude shows compressive fluctuations inside the jets which occur in anti‐phase with the density (see also Figure 1). Jets durations are 71 and 53 s respectively, which is shorter than those of J1 and J2. The plasma *β* is high through most of the interval, with values above 10, which drop a bit after J4. The value of the plasma beta during this interval was larger than for the plasma near J1 and J2, and *C*_*M*_ − *perp* was above 1 during the whole interval and also inside the jets, so we identify the fluctuations inside the jets as MMWs. It is interesting to note that ions inside J3 and J4 are modulated in pitch angle due to trapping in the troughs of the mirror mode structures. This is clearer inside J4 where more throughs occur. Ion heating inside the troughs (Soucek & Escoubet, 2011) leads to an anticorrelation between *T*_⊥_, *T*_‖_, and *B* magnitude (see also Figure 1). Ion trapping at intermediate pitch angles also occurs in the regions surrounding J3 and J4 where MMWs are present.

**Figure 9:**
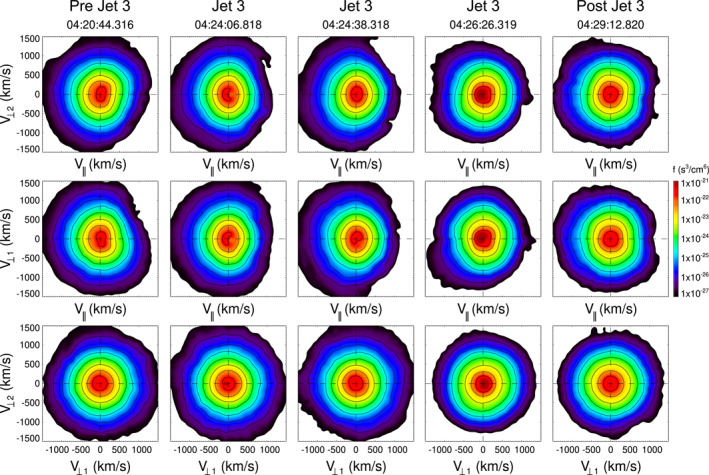
Velocity distribution functions near and inside J3. The format is as in Figure 4.

**Figure 10:**
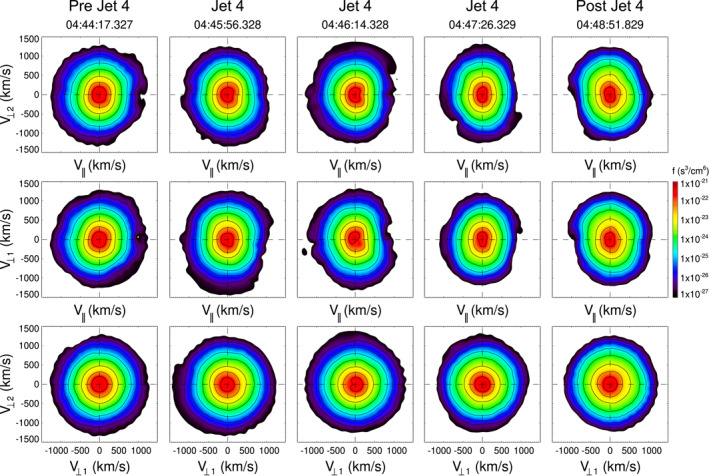
Velocity distribution functions near and inside J4. The format is as in Figure 4.

Figure 9 and 10 show that VDFs inside J3 and J4 are very similar to the distributions before and after each of them, in all cases a temperature anisotropy (*T*_⊥_ > *T*_‖_) is clearly observed, which is in contrast to ions inside J1 and J2.

### 2.4 Magnetic Fluctuations Inside and Outside of Jets

**Figure 11:**
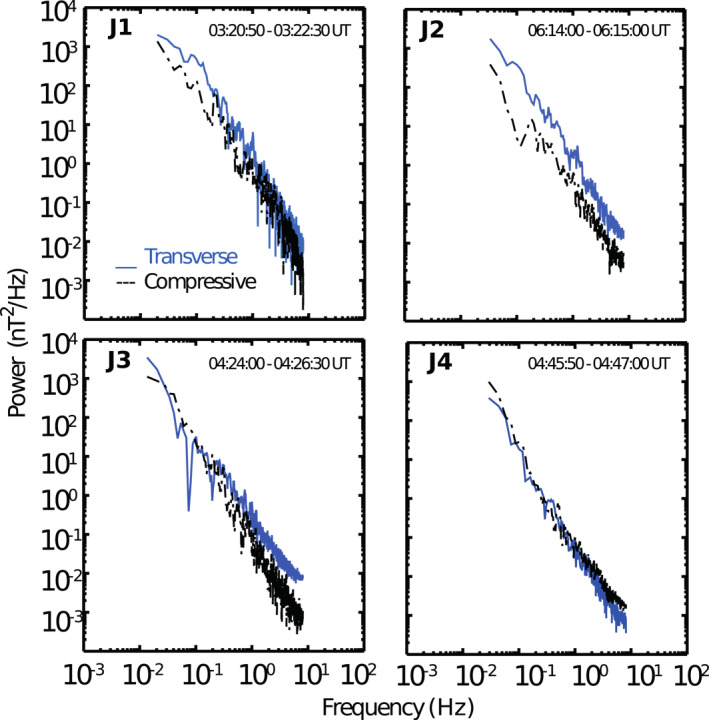
Power spectra for the fluctuations observed inside the four jets under study. The transverse (compressive) power is indicated by blue (black) lines. The time intervals correspond to the solid thick gray lines in Figures 3, 5, and 8.

Figure 11 shows transverse and compressive power spectra of waves inside the studied jets. The compressive power is defined from the total power *P*_*tot*_ which is the result of applying fast Fourier transformation to the total magnetic field *B*, while the transverse power is defined as ∣*P*_*x*_ + *P*_*y*_ + *P*_*z*_ − *P*_*tot*_∣, where *P*_*x*_, *P*_*y*_, *P*_*z*_, are the powers of *B*_*x*_, *B*_*y*_, and *B*_*z*_, respectively. It is possible to see that inside J1 and J2 the transverse component is the dominant one for low frequency fluctuations (*f* < 0.1 Hz); however, the fluctuations also have a strong compressive component. In contrast, the fluctuations inside J3 and J4 have opposite characteristics, they are more compressive with larger (J3) or equal (J4) power than the transverse component as expected for the mirror mode.

The regions surrounding J3 and J4 are populated by MMWs with peak‐like structure which are similar to the waves observed near J1, and in opposition to the hole‐like MMWs observed near J2. The association of jets with MMWs was discussed in a previous work (Kajdič et al., 2021) where enhancements in *P*_*dyn*_ occurred within isolated mirror mode structures and not for a train of MM. The MMWs observed inside J3 and J4 are different to the examples in Kajdič et al. (2021) where mirror modes were “hole‐like” with decrements in magnetic field magnitude, and occurred near the magnetopause where the instability criteria *C*_*M*_ − *perp* was satisfied only within them, and not in the surrounding plasma.

## 3 Discussion and Conclusions

In this work we have shown that structures identified as magnetosheath jets following the criteria of Archer and Horbury (2013) can coexist with MMWs, and that sometimes such waves can even be observed inside the jets. All the studied jets were detected nearby to the magnetopause in the quasi‐perpendicular magnetosheath. We have demonstrated that the micro‐structure of the studied events is variable, even though they were identified by the enhancement in dynamic pressure suggesting a completely different origin for each of them, and most probably not related to the mechanisms proposed by Hietala et al. (2012), Plaschke et al. (2013), Preisser, Blanco‐Cano, Kajdič, et al. (2020), Preisser, Blanco‐Cano, Trotta, et al. (2020), Karlsson et al. (2012), and Raptis, Karlsson, Vaivads, Pollock, et al. (2022) where phenomena related to the quasi‐parallel bow shock play a major role in jet formation.

The four studied events show that magnetosheath jets have a complex magnetic structure with variations in velocity, density and temperature. Table 1 summarizes jet properties in order to compare the structures and their different properties. All events have durations larger than the average 30 s reported for jets downstream of the quasi‐parallel shock (Archer & Horbury, 2013), and rather fit the durations found by Dmitriev et al. (2021) for larger scale jets. They show enhancements in speed and magnetic field magnitude. However, only J1 and J3 show enhancements in density. The field increment inside jets, as the ones shown here, has been reported by many authors (see Table 2 in Plaschke et al. (2018)). The field rotation inside J1 and J2 results in large ion pitch angle variations (with a range larger than 100°) which are not observed inside J3 and J4, where MMWs are found. Nonetheless, the ions inside J3 and J4 suffer some modulation due to trapping inside the magnetic troughs, which is clearer for J4.

**Table 1:** Properties for the Jets Studied: Duration in Seconds (*δt*), Variables Associated to Increments in Dynamic Pressure, Changes in Density, Magnetic Field, Temperature and Its Anisotropy, Pitch Angle, and Mirror Mode Condition Instability *C*_*M*_ − *perp*

Event	*δt* (s)	*P*_*dyn*_ increment	Density	*B*	*T*	*T*_⊥_/*T*_‖_	Pitch angle	*C*_*M*_
J1	110	*V*, *n*	Increases	Increases	Decreases	Variable	>100°	≤1
				*B*_*x*_ *B*_*y*_ *B*_*z*_ rotation				
J2	85	*V*	Decreases	Increases	Increases	<1	>100°	<1
				*B*_*x*_ rotation				
J3	71	*V*, *n*	Increases	Increases	Small decrement	>1	<70°	>1
				No rotation				
J4	53	*V*	No change	Increases	No change	>1	<50°	>1
				No rotation				

J1 shares some of the characteristics of jets reported often in the literature such as the decrement in temperature and broader ion energy spectra (Karlsson et al., 2018). In fact, the decrements occur in both *T*_⊥_ and *T*_‖_. As we noted earlier the ratio *T*_⊥_/*T*_‖_ remains above 1 during most of J1, with values close to 1 coinciding with the regions where magnetic fluctuations are larger, suggesting that wave particle interactions scatter the ions into more isotropic distributions. The fact that J1 is bounded by IMF rotational discontinuities indicates that its origin is due to a traveling foreshock (Kajdič et al., 2017) which has been transmitted downstream. Traveling foreshocks form due to magnetic flux tubes that are observed upstream of the quasi‐perpendicular bow shock but are connected to the quasi‐parallel section. A jet related to a traveling foreshock was discussed by Kajdič et al. (2021); however, some plasma properties of J1 differ from the jet studied by those authors, for which temperature increased, and the density had values below the average density of the surroundings.

The features of J2 indicate that this jet is compound of two structures, in the leading one, J2/1, the temperature increased while the density decreased. The temperature enhancement inside J2 is related to the hot magnetospheric material associated to the leading part of the jet (J2/1). The high value in temperature of the rear part is related to the moments calculation for a single VDF where two populations coexist. As we mentioned before, J2 exhibits properties of a FTE. In agreement with this interpretation, Archer and Horbury (2013) reported jets with density decrements and temperature increments as consistent with FTEs, containing hot plasma. Raptis et al. (2020) have also mentioned the possibility that a subset of the encapsulated jets is consistent with FTEs. It was mentioned in the detailed description of J2 that part of it resembles the properties of a FTE which was removed from its region of origin. This is supported by the fact that the guide field (*B*_*y*_ or *B*_*m*_) was high and sustained during the time of observation (Fear et al., 2005; Kawano & Russell, 1997). It is also possible that J2 was generated due to bursty reconnection driven by the thinning of the magnetopause caused by the impact of jets as suggested by Hietala et al. (2018) and Ng et al. (2021). The rear part J2/2 does not show the features of an FTE, although it is clear that two counterstreaming ion populations exist inside it. More work is needed to understand in detail the origin of jets similar to J2, as well as their substructure.

The temperature inside J3 shows a decrement during a very short interval, and does not change inside J4. These jets occur on a *B*_*z*_ south region, and their enhancement in *P*_*dyn*_ occurs mainly due to the increment of ion velocity. It is possible that these velocity enhancements are related to reconnection.

The difference in jet characteristics may not only depend on jet origin, but can also be influenced by the jet location within the magnetosheath. This is clearly the case for the values of the dynamic pressure which were smaller for J3 and J4, the jets observed in the subsolar region, in contrast to the higher values for J1 and J2 which are observed more to the flanks. A recent simulation study by Palmroth et al. (2021) shows that jet size and other parameters can change with propagation from the bow shock as they evolve. In particular, they showed that jets can be heated as they propagate to the magnetopause and interact with the surrounding magnetosheath plasma. This may explain why the J1, J3 and J4 do not show large decrements in temperature, being so close to the magnetopause. Future observational work should include multispacecraft analysis to understand jet evolution.

In regards to the VDFs associated with the studied jets, we find that ion distributions can be of different type. Single ion distributions are observed inside J1, J3, and J4. VDFs show some temperature anisotropy in all ion distributions associated with J3 and J4. These two jets were observed in a region where the plasma beta was higher than for J1 and J2, and this in combination with the temperature anisotropy results in a plasma highly unstable to the mirror mode instability. Ion trapping at magnetic troughs occurs inside J3 and J4, which is related to the “magnetic bottle” structure of MMWs. In contrast, counterstreaming ion distributions are observed inside J2, suggesting that this jet is linked to reconnection at an earlier time than when it was observed, in accordance with it resembling a FTE. Similar ion distributions have been observed inside FTEs (Petrinec et al., 2020) and observed near the magnetopause associated with reconnection (see e.g., Trattner et al., 2021). As pointed out by Raptis, Karlsson, Vaivads, Lindberg, et al. (2022) VDFs show evidence of two ion populations inside jets, as in the case of J2, and the calculation of separated moments for each distribution instead of moments for a single distribution where all populations are included would be more accurate to estimate jet properties.

Past work has shown that the magnetic fluctuations inside jets tend to be more transverse than compressive (Blanco‐Cano et al., 2020) as in the case of J1 and J2. To our knowledge, there is only one previous work linking jets to mirror mode structures (Kajdič et al., 2021) with large and isolated decrements in *B* magnitude that differ from the continuous mirror mode structures with peaks inside J3 and J4. According to Soucek et al. (2008), the different appearance of mirror mode shapes is related to plasma beta and to how unstable the mirror mode is in a specific region. Mirror structures in the form of magnetic holes are observed when plasma is mirror stable or marginally mirror unstable, with *β* < 5, while magnetic peaks are observed when the plasma is mirror unstable and tends to have higher values of beta (2–15). Figure 8 shows that J3 and J4 are immersed in a region where the mirror mode is unstable most of the time with *C*_*M*_ − *perp* > 1 and high plasma beta. In contrast, the jets discussed in Kajdič et al. (2021) were observed in a region where the mirror mode instability criteria was satisfied only inside them and not in the surrounding region. Therefore, MMWs inside J3 and J4 are still growing which is not the case for the jet discussed in Kajdič et al. (2021).

Our results show that the quasi‐perpendicular magnetosheath can be permeated by jets interacting with MMWs. MMWs are less often observed in the quasi‐parallel magnetosheath, so in such region jets may interact with other type of waves and magnetic fluctuations. Future work should investigate the interaction between jets and the waves (e.g., MMWs and ion cyclotron waves) as well as their joint effects on the magnetosphere (specially for jets surrounded by compressive fluctuations, which may enhance jet indentation of the magnetopause) and wave generation at the magnetosphere (see e.g., Archer et al., 2012; Plaschke et al., 2009; Wang et al., 2022).

Our study clearly demonstrates that magnetosheath jets have a more complex structure than previously thought ‐with even some defined substructure‐ and points out that VDF's characteristics should be taken into account more often as additional information regarding different jet formation mechanisms. We also conclude that some jets may form locally downstream of the quasi‐perpendicular shock, as a biproduct of reconnection, and not from an upstream structure. More work is needed to understand in detail how the various types of jets affect the magnetopause/magnetosphere due to their inherent pressure enhancements.

## Data Availability

We acknowledge MMS Science Data Center (https://lasp.colorado.edu/mms/sdc/public/) teams for access and visualization of the data. Data access and processing was done using SPEDAS V4.1, see Angelopoulos et al. (2019).
